# Emergence of *Escherichia coli* ST131 carrying carbapenemase genes, European Union/European Economic Area, August 2012 to May 2024

**DOI:** 10.2807/1560-7917.ES.2024.29.47.2400727

**Published:** 2024-11-21

**Authors:** Anke Kohlenberg, Olov Svartström, Petra Apfalter, Rainer Hartl, Pierre Bogaerts, Te-Din Huang, Katerina Chudejova, Lucia Malisova, Jessica Eisfeld, Mirco Sandfort, Anette M Hammerum, Louise Roer, Kati Räisänen, Laurent Dortet, Rémy A Bonnin, Ákos Tóth, Kinga Tóth, Christina Clarke, Martin Cormican, Algirdas Griškevičius, Kirstin Khonyongwa, Marie Meo, Baiba Niedre-Otomere, Reinis Vangravs, Antoni PA Hendrickx, Daan W Notermans, Ørjan Samuelsen, Manuela Caniça, Vera Manageiro, Vilhelm Müller, Barbro Mäkitalo, Urška Kramar, Mateja Pirs, Daniel Palm, Dominique L Monnet, Erik Alm, Marius Linkevicius

**Affiliations:** 1 European Centre for Disease Prevention and Control, Stockholm, Sweden; 2 Austrian National Reference Centre for Antimicrobial Resistance, Ordensklinikum Linz Elisabethinen, Linz, Austria; 3 National Reference Centre for Antimicrobic-Resistant Gram-Negative Bacilli, Laboratory of Microbiology, CHU UCL Namur, Yvoir, Belgium; 4 Department of Microbiology, Faculty of Medicine, University Hospital in Pilsen, Charles University, Pilsen, Czechia; 5 National Reference Laboratory for Antibiotics, National Institute of Public Health, Prague, Czechia; 6 Department of Microbiology, 3rd Faculty of Medicine, Charles University, University Hospital Kralovske Vinohrady and National Institute of Public Health, Prague, Czechia; 7 German National Reference Centre for Multidrug-resistant Gram-negative Bacteria, Department of Medical Microbiology, Ruhr-University Bochum, Bochum, Germany; 8 Department of Infectious Disease Epidemiology, Robert Koch Institute, Berlin, Germany; 9 National Reference Laboratory for Antimicrobial Resistance, Department of Bacteria, Parasites and Fungi, Statens Serum Institut, Copenhagen, Denmark; 10 Finnish Institute for Health and Welfare, Helsinki, Finland; 11 Associated French National Reference Centre for Antibiotic Resistance: Carbapenemase-Producing Enterobacteriaceae, Le Kremlin-Bicêtre, France; 12 Team “Resist” UMR1184 “Immunology of Viral, Auto-Immune, Hematological and Bacterial diseases (IMVA-HB)”, INSERM, Université Paris-Saclay, CEA, IHU Prometheus Faculty of Medicine, Le Kremlin-Bicêtre, France; 13 National Centre for Public Health and Pharmacy, Budapest, Hungary; 14 Galway Reference Laboratory Service, Galway University Hospital, Galway, Ireland; 15 School of Medicine, University of Galway, Galway, Ireland; 16 National Public Health Surveillance Laboratory, Vilnius, Lithuania; 17 Service Bactériologie-Mycologie-Antibiorésistance-Hygiène Hospitalière, Département de Microbiologie, Laboratoire National de Santé, Dudelange, Luxembourg; 18 National Microbiology Reference Laboratory of Latvia, Laboratory “Latvian Centre of Infectious Diseases”, Laboratory Service, Riga East University Hospital, Riga, Latvia; 19 Centre for Infectious Disease Control, National Institute for Public Health and the Environment (RIVM), Bilthoven, the Netherlands; 20 Norwegian National Advisory Unit on Detection of Antimicrobial Resistance, Department of Microbiology and Infection Control, University Hospital of North Norway, Tromsø, Norway; 21 National Reference Laboratory of Antibiotic Resistances and Healthcare Associated Infections, Department of Infectious Diseases, National Institute of Health Dr Ricardo Jorge, Lisbon, Portugal; 22 Public Health Agency of Sweden, Solna, Sweden; 23 National Laboratory of Health, Environment and Food, Centre for Medical Microbiology, Maribor, Slovenia; 24 Institute of Microbiology and Immunology, Faculty of Medicine, University of Ljubljana, Ljubljana, Slovenia

**Keywords:** Carbapenem-resistant Enterobacterales, carbapenemase, *Escherichia coli*, surveillance, whole genome sequencing, cross-border spread, antimicrobial resistance, antibiotics

## Abstract

Analysis of 594 isolates of *Escherichia coli* sequence type (ST)131 and its single locus variants carrying carbapenemase genes from 17 European Union/European Economic Area countries revealed acquisition of 18 carbapenemase variants, mainly in ST131 clades A and C. Most frequent were *bla*_OXA-244_ (n = 230) and *bla*_OXA-48_ (n = 224), detected in 14 and 12 countries, respectively. Isolates carrying *bla*_OXA-244_ have increased rapidly since 2021. The increasing detection of carbapenemase genes in the *E. coli* high-risk lineage ST131 is a public health concern.

In March 2024, the European Antimicrobial Resistance Genes Surveillance Network (EURGen-Net) operational contact points from Denmark contacted the European Centre for Disease Prevention and Control (ECDC) with concerns about increasing detection of OXA-244-producing *E. coli* ST131 in their country. Worldwide, *E. coli* is the pathogen associated with most deaths attributable to antimicrobial resistance [1]. Sequence type (ST)131 is a high-risk lineage of global distribution, frequently associated with multidrug resistance [2]. To date, there have been only few reports of carbapenemase gene-carrying *E. coli* ST131 isolates collected from human samples in European Union (EU)/European Economic Area (EEA) countries [3-5].

The aim of this investigation was to determine the epidemiological situation and genomic characteristics of *E. coli* ST131 and its single locus variants (SLVs) carrying carbapenemase genes in the EU/EEA based on the analysis of epidemiological and whole genome sequencing (WGS) data from national collections.

## Data collection and analysis

On 12 April 2024, the ECDC requested, via its EpiPulse platform, national reference laboratories that participate in EURGen-Net to provide WGS and epidemiological data of isolates of *E. coli* ST131 and its SLVs carrying carbapenemase genes. In response, 17 EU/EEA countries submitted 660 sequence datasets (500 short-read sets, 11 long-read sets, 116 short-read assemblies and 33 hybrid assemblies) from 627 isolates. After quality control and de-duplication, we analysed the sequences of 594 isolates carrying carbapenemase genes covering the period from August 2012 to May 2024 (Table 1).

**Table 1:** Isolates of *Escherichia coli* ST131 and its single locus variants carrying carbapenemase genes, by country, EU/EEA, August 2012–May 2024 (n = 594)

Carbapenemase gene	Number of isolates by country and period covered																	
	AT	BE	CZ	DE	DK	FI	FR	HU	IE	LT	LU	LV	NL	NO	PT	SE	SI	Total
	2022–2024	2023–2024	2021–2023	2022–2023	2014–2024	2021–2024	2019–2024	2023	2016–2024	2019–2023	2019–2024	2023	2012–2024	2012–2023	2016–2022	2017–2024	2022–2023	2012–2024
*bla*_OXA-244_	**8**	**3**	**1**	**32**	**25**	**3**	**85**	**1**	**14**	0	**2**	0	**28**	**6**	0	**21**	**1**	**230**
*bla*_OXA-48_	0	**2**	0	**1**	**9**	0	**82**	0	**101**	**1**	**1**	**1**	**13**	**3**	0	**9**	**1**	**224**
*bla*_NDM-1_	0	**2**	**2**	**1**	**3**	**1**	**7**	**1**	**4**	0	0	0	**4**	**2**	**3**	0	**1**	**31**
*bla*_NDM-5_	0	0	0	**1**	**3**	0	**8**	0	**2**	0	**1**	0	**3**	0	0	**2**	0	**20**
*bla*_KPC-2_	0	0	0	**1**	0	0	**1**	0	**3**	**11**	0	0	**1**	0	0	**2**	0	**19**
*bla*_OXA-181_	0	0	0	**1**	**3**	0	**8**	0	**3**	0	0	0	0	**1**	0	0	0	**16**
*bla*_KPC-31_^a^	0	0	0	0	0	0	**1**	0	0	0	0	0	0	0	**14**	0	0	**15**
*bla*_VIM-1_	0	**1**	0	**1**	0	0	**3**	0	0	0	0	0	**9**	0	0	0	0	**14**
*bla*_KPC-3_	0	**1**	0	**1**	**1**	**2**	**2**	0	**1**	0	**1**	**1**	0	0	**1**	**1**	0	**12**
*bla*_NDM-7_	0	0	0	0	0	0	**2**	0	0	0	0	0	0	0	0	0	0	**2**
*bla*_OXA-484_	0	**1**	0	0	0	0	**1**	0	0	0	0	0	0	0	0	0	0	**2**
*bla*_NDM-18_	0	0	0	**1**	0	0	0	0	0	0	0	0	0	0	0	0	0	**1**
*bla*_VIM-4_	0	0	0	0	0	0	**1**	0	0	0	0	0	0	0	0	0	0	**1**
*bla*_KPC-53_	0	0	0	0	0	0	0	0	0	0	0	0	0	0	**1**	0	0	**1**
*bla*_KPC-225_	0	0	0	0	0	0	0	0	0	0	0	0	0	0	**1**	0	0	**1**
*bla*_OXA-204_	0	0	0	0	0	0	**1**	0	0	0	0	0	0	0	0	0	0	**1**
*bla*_OXA-232_	0	0	0	0	0	0	0	0	0	0	0	0	**1**	0	0	0	0	**1**
*bla*_OXA-244*_ *^b^*	0	0	0	0	0	0	0	0	0	0	0	0	**1**	0	0	0	0	**1**
*bla*_NDM-5_/*bla*_OXA-232_	0	0	0	0	0	0	0	0	0	0	0	0	0	**1**	0	0	0	**1**
*bla*_NDM-5_/*bla*_OXA-244_	0	0	0	0	0	0	0	0	**1**	0	0	0	0	0	0	0	0	**1**
**Total**	**8**	**10**	**3**	**40**	**44**	**6**	**202**	**2**	**129**	**12**	**5**	**2**	**60**	**13**	**20**	**35**	**3**	**594**

AT: Austria; BE: Belgium; CZ: Czechia; DE: Germany; DK: Denmark; EU/EEA: European Union/European Economic Area; FI: Finland; FR: France; HU: Hungary; IE: Ireland; LT: Lithuania; LU: Luxembourg; LV: Latvia; NL: the Netherlands; NO: Norway; PT: Portugal; SE: Sweden; SI: Slovenia; ST: sequence type.

^a^ *bla*_KPC-31_ listed in the Beta-Lactamase DataBase (http://bldb.eu, accessed 28 October 2024) as carbapenemase, but reported in literature as extended spectrum beta-lactamase not conferring carbapenem resistance [24].

*^b^bla*_OXA-244*_ represents the new *bla*_OXA-48-like_ variant with C100T substitution leading to H34T amino acid change.

Numbers in bold indicate detection of ≥ 1 isolate.

Short-reads were assembled using SPAdes v3.15.5 [6] and long-reads using Flye v2.9.4 [7]. Alleles were called using ChewBBACA v3.3.4 [8] and the *Escherichia*/*Shigella* core genome multilocus sequence typing (cgMLST) scheme from EnteroBase [9]. Serotyping was performed using the *E. coli* analysis plugin of BioNumerics 7.6.3 (Applied Maths NV/bioMérieux). We assigned ST with the Center for Genomic Epidemiology (CGE) MLST v2.0.9 tool [10], using the 7-gene MLST scheme by Achtman [11]. We used the CGE FimTyper to assign the type 1 fimbriae adhesin *fimH* allele [12]. We identified antimicrobial resistance genes with ResFinder v4.1.11 with default settings [13]. Clusters were assigned using single-linkage clustering with a cut-off of 10 allelic differences [14].

## Distribution of carbapenemase genes

We detected 18 different carbapenemase genes in the *E. coli* ST131 isolates, including ST131 SLVs. Two carbapenemase genes, *bla*_OXA-244_ (n = 230) and *bla*_OXA-48_ (n = 224), together accounted for 76% of the isolates (Table 1), followed by *bla*_NDM-1_ in 31 (5%) and *bla*_NDM-5_ in 20 (3%) isolates. All other carbapenemase genes were detected in fewer than 20 isolates (Table 1). The isolates carrying *bla*_OXA-244_ were detected in 14 countries. Isolates carrying *bla*_OXA-48_ were detected in 12 countries, although most originated from France and Ireland. Despite the much smaller numbers of isolates carrying *bla*_NDM-1_ or *bla*_NDM-5_, these isolates were also detected in 12 and seven countries, respectively.

**Figure 1:**
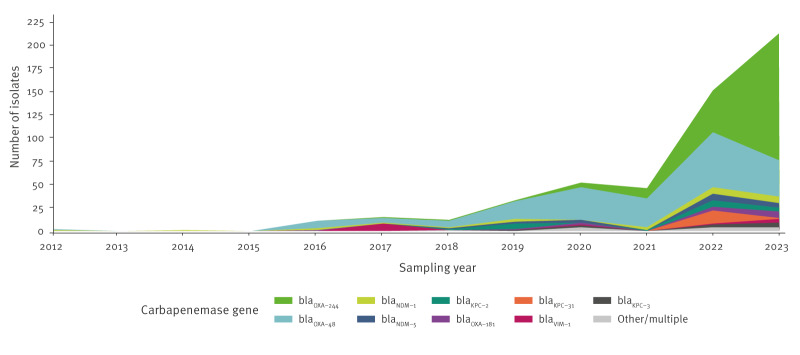
Number of *Escherichia coli* ST131 isolates, including its single locus variants^a^, carrying carbapenemase genes, by year, EU/EEA, 2012–2023^b^ (n = 535)

While *E. coli* ST131 isolates carrying *bla*_OXA-48_ appeared earlier than isolates with *bla*_OXA-244_ (2012 vs 2017), their frequency of detection increased only moderately over time, with a peak in 2022 followed by a small decrease in 2023 (Figure 1).

EU/EEA: European Union/European Economic Area.

^a^ Detected single locus variants included ST8420, ST11358, ST11362, ST11672, ST13133 and ST13730.

^b^ Three isolates without information on year of sampling and 56 isolates from 2024 (as that year was incomplete at time of sampling) were excluded.

In contrast, detection of isolates carrying *bla*_OXA-244_ increased sharply between 2021 and 2023. In addition, we observed an increasing diversity of carbapenemase (including metallo-beta-lactamase) genes over time, although without a clear trend for any of the six metallo-beta-lactamase genes detected in this analysis.

## Epidemiological and microbiological characteristics

Based on the varying frequency and time trends, we divided the isolates into three groups for further analysis: Group 1: *E. coli* ST131 isolates, including ST131 SLVs, carrying *bla*_OXA-244_; Group 2: isolates carrying *bla*_OXA-48_; and Group 3: isolates carrying other carbapenemase genes (Table 2). In the epidemiological analysis, Group 1 stood out with a high proportion of female patients, a relatively low median age, the frequent detection of isolates from urine samples, and slightly more frequent documentation of travel outside the EU/EEA within 12 months before detection (Table 2).

**Table 2:** Epidemiological and genomic characteristics of carbapenemase genes carrying isolates of *Escherichia coli* ST131 and its single locus variants, EU/EEA, August 2012–May 2024 (n = 594)

Characteristic	Group 1: *bla*_OXA-244_ n = 230		Group 2: *bla*_OXA-48_ n = 224		Group 3: other n = 140	
	n	%	n	%	n	%
Median age (years)	57		77		70	
Sex						
Male	69	30	81	36	51	36
Female	135	59	82	37	57	41
Not available	26	11	61	27	32	23
Type of sample						
Urine	115	50	58	26	57	41
Rectal/faeces	23	10	94	42	17	12
Blood	6	3	5	2	8	6
Other	28	12	11	5	15	11
Not available	58	25	56	25	43	31
Travel outside the EU/EEA in the past 12 months						
Yes	35	15	5	2	8	6
No	16	7	3	1	7	5
Not available	179	78	216	96	125	89
Destinations (number of travel links to respective destination)	Türkiye (17), Egypt (8), Algeria (3), Morocco (2), Guinea (1), India (1), Jordan (1), Senegal (1), Tunisia (1)		Iran (1), Syria (1), Thailand (1), Venezuela (1), Viet Nam (1)		Ukraine (2), Albania (1), Colombia (1), Morocco (1), India (1), Somalia (1), Senegal (1)	
Serotype						
O16:H5	148	64	80	36	50	36
O25:H4	73	32	127	57	84	60
Other	1	0	4	2	1	1
Unknown	8	3	13	6	5	4
Sequence type						
131	182	79	209	93	138	99
13730	42	18	0	0	0	0
Other	6	3	15	7	2	1
*fimH* allele^a^						
*fimH*41 (Clade A marker)	153	67	85	38	51	36
*fimH*30 (Clade C marker)	75	33	115	51	63	45
*fimH*22 (Clade B marker)	0	0	9	4	16	11
Other	2	1	10	4	10	7
Absent	0	0	5	2	0	0
Fluoroquinolone resistance mutation(s)						
GyrA S83L; ParC S80I, E84V	96	42	1	0	1	1
GyrA S83L, D87N; ParC S80I, E84V	52	23	93	42	69	49
GyrA S83L only	26	11	42	19	29	21
GyrA S83L, D87N; ParC S80I	6	3	4	2	5	4
Other	13	6	3	1	1	1
Absent	37	16	81	36	35	25
ESBL gene(s)						
*bla*_CTX-M-15_	172	75	47	21	44	31
*bla*_CTX-M-27_	7	3	20	9	28	20
*bla*_SHV-12_	0	0	1	0	16	11
Other/multiple	3	1	5	2	12	9
Absent	48	21	151	67	40	29

ESBL: extended-spectrum beta-lactamase; EU/EEA: European Union/European Economic Area; SLV: single locus variant; ST: sequence type.

Genomic characteristics present in less than 10 isolates are grouped into the category ‘other’. Category unknown for serotype signifies that either O antigen or H antigen type, or both, were not assigned by BioNumerics 7.6.3.

^a^ Alleles of *fimH* correlate with major clades of *E. coli* ST131. Of 253 isolates carrying *fimH*30, 251 were assigned to subclades C0 (n = 62), C1 (n = 57) and C2 (n = 132) based on the topology of the cgMLST phylogeny combined with absence (C0) or presence (C1/C2) of mutations in GyrA (S83L, D87N) and ParC (S80I, E84V) as well as detection of specific *bla*_CTX-M_ genes, i.e. *bla*_CTX-M-27_ indicative of C1 or *bla*_CTX-M-15_ of C2.

Most Group 1 isolates belonged to serotype O16:H5, while the majority of group 2 and 3 isolates were of serotype O25:H4 (Table 2). Single locus variants of *E. coli* ST131 were most frequent among Group 1 isolates, all of which belonged to ST13730 (Table 2). Typing of *fimH* showed that the most frequent *fimH* allele in Group 1 was the clade A marker *fimH*41 followed by *fimH*30 indicative of clade C. In contrast, in Groups 2 and 3, more isolates were carrying *fimH*30 than *fimH*41 (Table 2). Of the 251 isolates with *fimH30* in clade C, 132 were assigned to subclade C2, followed by C0 (n = 62) and C1 (n = 57). Resistance markers also varied by group, e.g. the extended-spectrum beta-lactamase (ESBL) gene *bla*_CTX-M-15_ was markedly more frequent in Group 1 than in Group 2 and 3 isolates. In addition, of 263 isolates co-carrying *bla*_CTX-M-15_, more than half (n = 150) had the clade A marker *fimH*41, followed by isolates with clade C marker *fimH*30 (n = 110), and clade B marker *fimH*22 (n = 2) and *fimH*27 (n = 1). We observed similar variation between groups for fluoroquinolone resistance mutations (Table 2).

## Genomic relatedness

**Figure 2:**
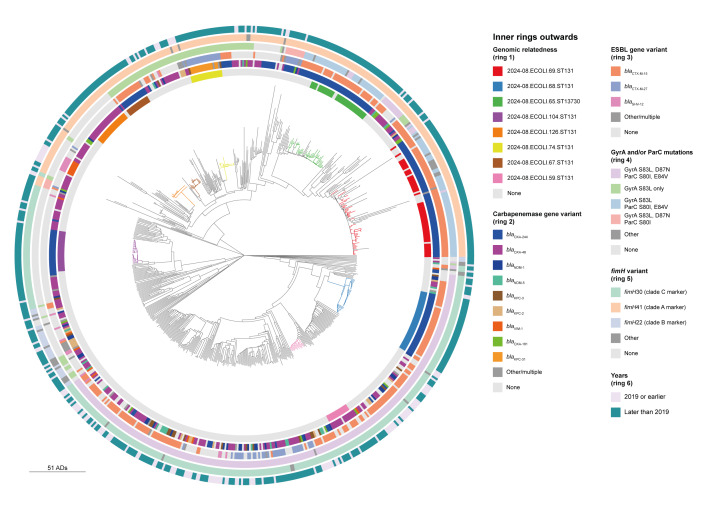
Phylogenetic tree of *Escherichia coli* ST131 isolates, including its single locus variants included in the genomic relatedness analysis, EU/EEA and outside, 2005–2024 (n = 691)

ADs: allelic differences; cgMLST: core genome multilocus sequence typing; ESBL: extended-spectrum beta-lactamase; EU/EEA: European Union/European Economic Area.

For the investigation of genomic relatedness, we added 93 isolates from ECDC surveys and investigations and four control isolates from the National Center for Biotechnology Information (NCBI) representing clades A, B and C, resulting in a dataset of 691 isolates. Eight larger clusters (n ≥ 10 isolates) were detected, including six clusters with isolates from 2024. Isolates in these eight clusters were carrying *bla*_OXA-244_ (five clusters) followed by *bla*_OXA-48_ (two clusters) and *bla*_KPC_ variants (one cluster) (Figure 2).

The phylogenetic tree was constructed based on cgMLST using neighbour joining algorithm. Of note, a cgMLST-based phylogeny has limitations on resolving *E. coli* ST131 population structure and division into clades. Categories in each ring are arranged in decreasing order of frequency and represent findings from more than 10 isolates. The eight largest clusters with isolates within 10 ADs in the tree are coloured following the ring 1 legend.

Isolates carrying *bla*_OXA-244_ formed multi-country clusters, while clusters of *bla*_OXA-48_-carrying isolates were predominantly detected within one country, e.g. France or Ireland. The eight clusters belonged to *E. coli* ST131 clade A (five clusters) or subclades C0 (one cluster) and C2 (two clusters). From a cladistic point of view, two clusters within clade A contained additional isolates that did not meet the single-linkage cluster definition. These were not counted in the cluster statistics. Four of the five clusters carrying *bla*_OXA-244_ included at least one isolate for which available hybrid assemblies confirmed the location of *bla*_OXA-244_ on the chromosome. A detailed description of the eight large clusters can be found in the Supplementary Figure.

## Discussion

We report the emergence of *E. coli* ST131 carrying carbapenemase genes based on genomic and epidemiological data from 17 EU/EEA countries. We observed an increasing frequency of detections and diversity of carbapenemase genes from 2012 to 2024. Furthermore, we detected considerable heterogeneity in the geographical distribution and speed of spread of specific carbapenemase genes, in particular for the recent rapid emergence of ST131 isolates carrying chromosomally localised *bla*_OXA-244_ associated with large multi-country clusters.

The increasing detection of carbapenemase genes in *E. coli* ST131 documented in this study is of concern because *E. coli* can cause a variety of infections in healthcare and community settings, frequently urinary tract infections, but also including bloodstream infection [15]. Worldwide, *E. coli* ST131 is the predominant extraintestinal pathogenic *E. coli* (ExPEC) lineage. It has been strongly associated with the global dissemination of the *bla*_CTX-M-15_ ESBL gene [2], and there is a high risk that it can play a similar role for the global spread of carbapenemase genes.

While the pooling of data from 17 EU/EEA countries facilitated early detection of this emerging resistance pattern, our study was based on routine national surveillance with differences in sample collection protocols, coverage and data completeness (in particular related to missing data for sample type and travel history), which is a limitation. Nevertheless, the age, sex, sample type and travel history distribution of isolates carrying *bla*_OXA-244_ suggest a potential association with community-acquired urinary tract infections (UTI), although this would need to be confirmed by studies with harmonised sampling. Of note, *E. coli* carrying *bla*_OXA-244_ often do not grow on screening media for carbapenemase-producing Enterobacterales (CPE) [16] and are most likely under-detected. The apparent association of *E. coli* ST131 carrying *bla*_OXA-244_ with community-acquired UTIs might therefore only represent the tip of the iceberg in terms of patient colonisation in the community.

Previous global surveys of carbapenemase-producing *E. coli* covering different geographical areas and time periods have identified only few *E. coli* ST131 isolates carrying *bla*_OXA-48_ and none carrying *bla*_OXA-244_ [17,18]. Although *E. coli* ST131 clade C has been reported as the primary contributor to fluoroquinolone resistance and the spread of ESBL genes globally [19], clade A had a higher rate of increase in estimated effective population size in a longitudinal survey in Norway [20]. Our analysis found large multi-country clusters within clade A and C. Clusters in clade A, where the cluster definition did not capture the full diversity within the cluster-defining branch, may represent clonal expansion over time rather than recent transmission events. We also found frequent co-carriage of *bla*_CTX-M-15_ in clade A, although this clade was previously described in some but not all studies as rarely carrying ESBL genes [19-21]. However, co-carriage of *bla*_CTX-M_ genes may differ by time, geographical region and selection criteria for the studied isolates. It is a limitation of this investigation that we did not analyse a random population of *E. coli* ST131 but isolates pre-selected for carriage of carbapenemase genes, which probably resulted in an isolate collection with a higher likelihood for co-carriage of other resistance markers.

As ExPEC has been identified in various non-human reservoirs and can be transmitted via the faecal-oral, household, sexual or food-borne routes [15], it is difficult to control its spread within the human population. There are now various examples of increasing dissemination of carbapenemase-producing ExPEC in the EU/EEA, such as *E. coli* ST131 as shown in this study, but also *E. coli* ST167, ST405, ST410 and ST648 carrying *bla*_NDM-5_ [22] and *E. coli* ST38 carrying *bla*_OXA-244_ [23].

## Conclusion

The increasing detection of *E. coli* ST131 carrying carbapenemase genes with potential community acquisition and dissemination sends another warning about the worsening epidemiological CPE situation in the EU/EEA. Further spread of *E. coli* carrying carbapenemase genes would mean that carbapenems could no longer be consistently effective for empiric treatment of severe *E. coli* infections. Urgent public health action is required to improve control of CPE in the EU/EEA and worldwide.

## Supplementary Data

447000 Supplementary Figure

## Supplementary Data

50000 Supplementary Table
