# Supplementary material for: Emergence of Escherichia coli ST131 carrying carbapenemase genes, European Union/European Economic Area, August 2012 to May 2024
Source: Euro Surveill. 2024 Nov 21;29(47):2400727. doi: 10.2807/1560-7917.ES.2024.29.47.2400727 (PMC11583312; doi:10.2807/1560-7917.ES.2024.29.47.2400727)
Supplement: Supplementary Figure [file 24-00727_KOHLENBERG_Supplementary_Figure.pdf]

Emergence of *Escherichia coli* carrying carbapenemase genes, European Union/European Economic Area, August 2012 to May 2024

Supplementary material

This supplementary material is hosted by *Eurosurveillance* as supporting information alongside the article “Emergence of *Escherichia coli* carrying carbapenemase genes, European Union/European Economic Area, August 2012 to May 2024”, on behalf of the authors, who remain responsible for the accuracy and appropriateness of the content. The same standards for ethics, copyright, attributions and permissions as for the article apply. Supplements are not edited by *Eurosurveillance* and the journal is not responsible for the maintenance of any links or email addresses provided therein.

Supplementary Figure. Time distribution of isolates of *Escherichia coli* ST131, including its single locus variants (SLVs) within clusters by reporting country and cluster, 2014-2024[a] (n = 350)

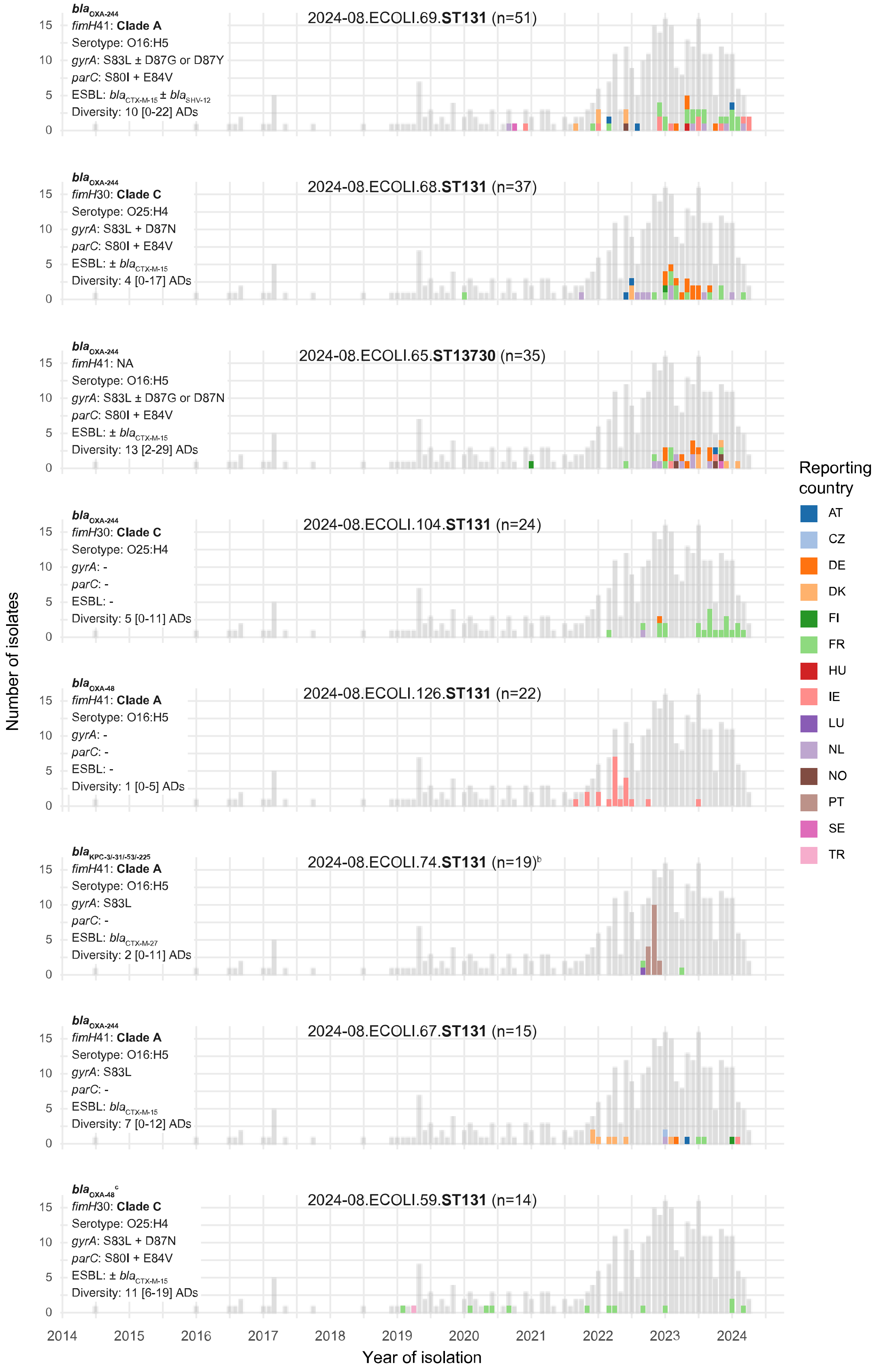

AT: Austria; CZ: Czechia; DE: Germany; DK: Denmark; FI: Finland; FR: France; HU: Hungary; IE: Ireland; LU: Luxembourg; NL: the Netherlands; NO: Norway; PT: Portugal; SE: Sweden; TR: Türkiye. AD: allelic difference; NA: not applicable, even though the ST13730 isolates branch out within ST131 clade A; ST: sequence type.

The plot includes only isolates within clusters (grey) with the largest clusters containing more than ten isolates coloured by reporting country in panels above. Within cluster diversity is described as median allelic differences and range.

[a] Three isolates without information on year of sampling were not marked in the plots.

[b] bla_{KPC-31} listed in Beta-Lactamase DataBase (http://bldb.eu/, accessed 28 October 2024) as carbapenemase, but reported in literature as extended spectrum beta-lactamase not conferring carbapenem resistance [Haidar G, Clancy CJ, Shields RK, Hao B, Cheng S, Nguyen MH. Mutations in bla_{KPC-3} that confer ceftazidime-avibactam resistance encode novel KPC-3 variants that function as extended-spectrum β-lactamases. Antimicrob Agents Chemother. 2017;61(5):e02534-16. http://dx.doi.org/10.1128/AAC.02534-16 PMID:28223379].

[c] An isolate from Türkiye clustered within 2024-08.ECOLI.59.ST131, but did not carry bla_{OXA-48}.
